# Compound Microstructures and Wax Layer of Beetle Elytral Surfaces and Their Influence on Wetting Properties

**DOI:** 10.1371/journal.pone.0046710

**Published:** 2012-10-04

**Authors:** Mingxia Sun, Aiping Liang, Gregory S. Watson, Jolanta A. Watson, Yongmei Zheng, Lei Jiang

**Affiliations:** 1 Key Laboratory of the Zoological Systematics and Evolution, Institute of Zoology, Chinese Academy of Sciences, Beijing, People’s Republic of China; 2 Centre for Biodiscovery and Molecular Development of Therapeutics, James Cook University, Townsville, Australia; 3 School of Pharmacy and Molecular Sciences, James Cook University, Townsville, Australia; 4 Key Laboratory of Bio-Inspired Smart Interfacial Science and Technology of Ministry of Education, School of Chemistry and Environment, Beihang University, Beijing, People’s Republic of China; 5 Center of Molecular Sciences, Institute of Chemistry, Chinese Academy of Sciences, Beijing, People’s Republic of China; University of Akron, United States of America

## Abstract

A beetles’ first line of defense against environmental hazards is their mesothoracic elytra – rigid, protective forewings. In order to study the interaction of these wings with water, the surface microstructures of various beetles’ elytra were observed by Environment Scanning Electron Microscopy (ESEM) and Atomic Force Microscopy (AFM). Chemistry components were ascertained using X-ray photoelectron spectroscopy (XPS). All the beetles of various habitats (including desert, plant, dung, land and water) exhibited compound microstructures on their elytra. The wetting properties of these elytra were identified using an optical contact angle meter. In general the native elytra exhibited hydrophilic or weak hydrophobic properties with contact angles (CAs) ranging from 47.5° to 109.1°. After treatment with chloroform, the CAs all increased on the rougher elytral surfaces. The presence of wax is not the only determinant of hydrophobic properties, but rather a combination with microscopic structures found on the surfaces. Irregularities and the presence or absence of tiny cracks, hairs (or setae), pores and protrusions are important factors which influence the wetting properties. Rougher elytral surfaces tended to present a stronger hydrophobicity. Effects on hydrophobicity, such as surface microstructures, chemistry, environment and aging (referring to the time after emergence), are also included and discussed. Our results also provide insights into the motion of water droplets when in contact with beetle elytra.

## Introduction

Hydrophobicity and hydrophilicity of solid surfaces have been researched extensively not only from a classical theory perspective [1]–[3], but also in terms of potential applications [3], [4]. Biological surfaces have received considerable interest with both flora and fauna studies [5]–[11]. Some biological micro/nano-structuring has been shown to enhance the wetting properties of the surface. An example is the lotus leaf, the superhydrophobic consequence of this feature is termed the “Lotus-effect” [12], where the rolling motion of water droplets collects surface contaminants resting on micro-papillae and nanoscale branchlike structures. Differing from the lotus, the red rose “Petal-effect” [13], [14] demonstrates superhydrophobicity with a high adhesive force of droplets with the micro/nano structures.

The wettability of insect cuticle has received little attention compared with the large number of species and diverse structuring that exists. Holdgate [15] has characterized four major groups of insects in relation to their water wetting properties. These include terrestrial and aquatic species which can comprise of smooth and rough surface cuticles. One of the interesting groups includes the terrestrial and semi-aquatic species whose surfaces are very rough or covered with hair piles. They have very high advancing and receding contact angles (CAs), often over 150°, which generally indicates low adhesion to water. These adaptations are more often structural rather than chemical since many insects already have chemistry which is at the near upper limit for smooth surfaces.

The wings of insects often display an intricate structuring as they represent large surface areas where contamination from water can have serious consequences (e.g., immobilization or reduced capacity to fly). Insects can be divided into two groups based on a quotient of wing surface area to body mass. Taxa with a high quotient (often insects with large wings) generally possess unwettable wings and show high particle removal due to the rolling motion of water drops [16]. A low quotient (e.g., small winged insects such as house flies and bees) tends to present more hydrophilic properties [16]. Certain insect species such as butterflies [17], [18], water striders [19], [20], lacewing [21], [22], termites [23], craneflies [24], and cicadas [25]–[28], all present wings or legs which are (super)hydrophobic with micro- and often underlying nano-structures present in each case. Recently, some of these biosurface architectures have been successfully fabricated using a combination of different techniques [29]–[31].

Coleopteran is the largest group of insects. A feature of some species in this group is a hard protective layer called the elytra. This hard exterior of a beetle protects the inner soft wing from damage. It has been found certain species have properties of reduced adhesion [32], differences in mechanical properties (e.g., for the folded part, away from the body, a lower hardness and Young's modulus has been measured [33]) and specific coloring mechanisms (e.g., structuring of multilayer reflectors, three-dimensional photonic crystals, diffraction gratings [34]). While various features of the elytra have been studied, the wettability has received little attention [15], [35], even though the cuticle on these regions may present a higher susceptibility due to reduced motion for removal of water (i.e., elytra display limited rapid motion compared to wing action).

In this paper various adult beetles of various habitats including desert, plant, dung, land and water, were selected to explore wettable properties. By comparing the effects of microstructure and chemistry on hydrophobicity, and investigating pore (secretion channels) arrangement and chemical composition on the elytral surfaces, we can provide a reference for studies of wettability under different environments.

## Materials and Methods

### Ethics Statement

No specific permits were required for the described field studies and the localities where the studied specimens were collected are not privately-owned or protected in any way.

### Insects and Preparation

Eleven species of Coleopteran were procured in different provinces of China on different dates (Table 1). The adult beetles are shown in Fig. S1: desert beetles, *Anatolica kulzeri* and *Mantichorula semenowi*; leaf dweller, *Anomala* sp.; dung beetles, *Catharsius molossus*, *Catharsius* sp. and *Gymnopleurus* sp.; semi-aquatic beetles, *Sominella macrocnemia* and *Amphizoa sinica*; and aquatic beetles, *Hydrophilus dauricus*, *Hydaticus grammicus* and *Hydrochara* sp. Elytra of individual beetles were cleaned with flowing deionized water to remove external contaminants. Some of samples were also rinsed with chloroform using a micro injector of 10 µL at a speed of 0.5 ∼ 1 µL s^−1^ for approx. 1 min. Finally, all the samples were sectioned into squares of ca. 0.5×0.5 cm^2^ from the central flat wing sections using scissors prior to experimentation.

**Table 1:** Data of the 11 species of beetles studied - collection dates, habitat, contact angles (CAs) and the roughness average (Ra) on the elytral surfaces before/after flowing chloroform treatment.

Species(Fig. S1 label)	Dates(D. M. Y)	CAs (°)Before/After	CA(°)	Habitat	Ra(×10^2 ^nm)Before/After
*Anatolica kulzeri* (a)	30.06.2007	47.5/80.7	33.2	desert	36/64
*Mantichorula semenowi* (b)	30.06.2007	78.8/105.8	27.0	desert	31/91
*Anomala* sp. (c)	6.09.2004	89.9/112.2	22.3	plant	65/162
*Catharsius molossus* (d)	15.09.1993	106.9/114.8	7.9	dung	141/154
*Catharsius* sp. (e)	6.06.1981	93.9/105.0	11.1	dung	47/94
*Gymnopleurus* sp. (f)	4.08.1983	71.3/112.4	41.1	dung	104/132
*Sominella macrocnemia* (g)	6.06.1954	107.5/122.1	14.6	s/aquatic	74/135
*Amphizoa sinica* (h)	17.07.1991	109.1/110.0	0.9	s/aquatic	118/141
*Hydrophilus dauricus* (i)	11.08.2007	66.2/97.6	31.4	aquatic	37/104
*Hydaticus grammicus* (j)	13.08.2007	79.9/105.1	25.2	aquatic	49/79
*Hydrochara* sp. (k)	13.08.2007	88.3/103.1	14.8	aquatic	52/89

### Microstructure Observation and CA Measurements

The methods of microstructure observation and CA measurements on the elytral surfaces before and after chloroform treatment have been described previously [26]. The parameters of microstructures were measured using the software ImageJ, and all CAs are shown in Table 1.

### Chemistry Components Analysis

The chemical components of the native elytra surfaces of six species were ascertained using X-ray photoelectron spectroscopy (Sigma Probe, Thermo VG-Scientific, ESCALAB 250 Thermo Fisher, England). The samples were fixed onto the stage using conductive adhesive. Experimental conditions were as follows: monochrome, anode target - Al; the energy resolution of the full-spectrum analysis - 100 eV, stepwise - 1eV; the energy resolution of the narrow-band spectrum analysis - 20eV, stepwise - 0.1eV; X-launched area - 500 µm; the pressure in the vacuum chamber - 1×10^–9 ^mBar.

### Surfaces Roughness Determination

The native surface roughness of three species, two aquatic (*H. dauricus* and *Hydrochara* sp.) and one semi-aquatic (*A. sinica*) beetles, were obtained using Atomic Force Microscopy (AFM) (FastScan, Bruker, America). The scanning range was 20 µm^2^, with roughness values referring to the root mean square (RMS) roughness.

The surface roughness data of the other samples, including the native and treated elytra, were obtained using the software Gwyddion based on SEM images, and all the roughness average (Ra) data are listed in Table 1.

## Results

### Microstructure Observation

The 11 beetles involved in this study include two desert beetles (Figs. S1a, b), four terrestrial (Fig. S1c – leaf dwelling, Figs. S1d–f – dung beetles), two semi-aquatic (Figs. S1g, h) and three aquatic (Figs. S1i–k) species. A diversity of elytral surface microstructures was observed.

**Figure 1:**
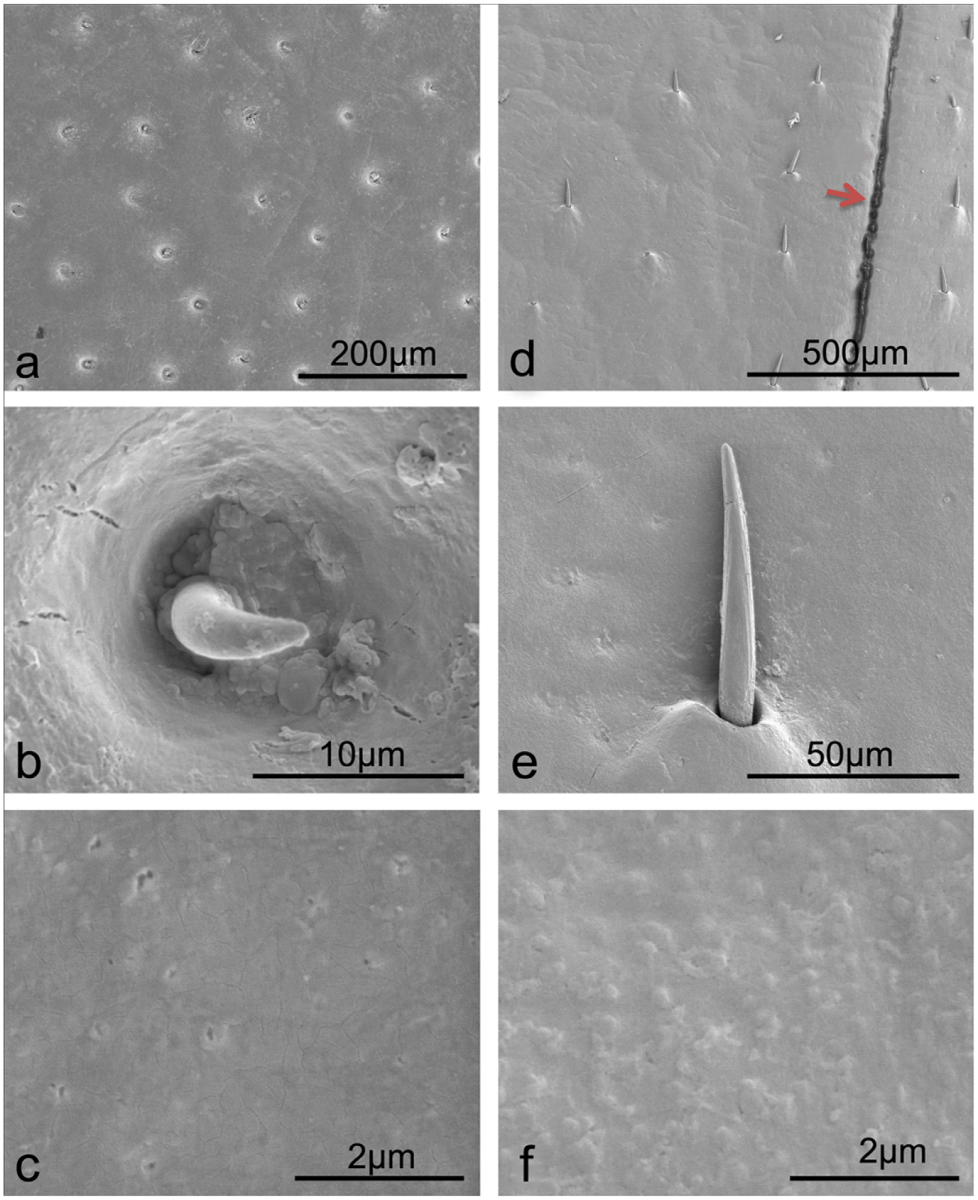
SEM images of microstructures and setae on the two desert beetle elytral surfaces. a–c. *A. kulzeri*; d–f. *M. semenowi*. The red arrow in (d) highlights the joint of two elytra.

Both of the desert beetles (*A. kulzeri* and *M. semenowi*) possess setae (small hairs) on the native elytral surfaces (Figs. 1a, d, respectively). The setae of *A. kulzeri* (length = 7.67±0.16 µm, diameter at centre = 2.88±0.17 µm) are located in small pits (Fig. 1b), where they protrude perpendicular to the elytra surface, whereas the setae of *M. semenowi* (length = 70.59±4.80 µm, diameter at centre = 7.97±0.20 µm) lay flat to the elytra surface with the base of the epidermis being slightly swollen (Fig. 1e). Secretion pores are less pronounced in *M. semenowi* than *A. kulzeri* as seen in Figs. 1f and c, respectively. SEM images in Fig. 1c reveal nanometer sized pores (or pits) whereas there is little evidence of these in Fig. 1f. This may indicate that there is a thicker layer of secretions from the cuticle cells of *M. semenowi*. Interestingly, the topography of *M. semenowi* setae also reveals nanochannels running along the hair shaft.

**Figure 2:**
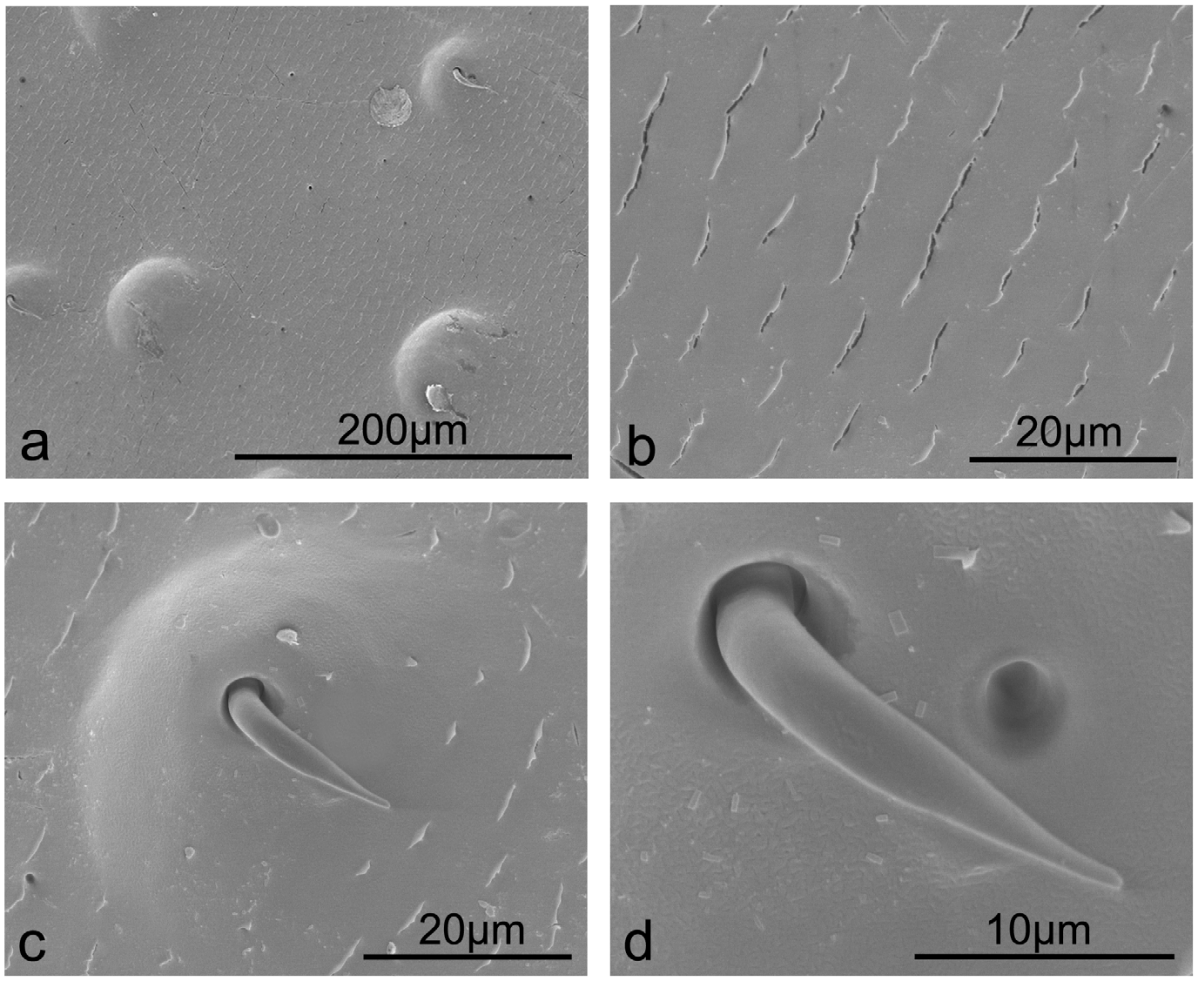
SEM images revealing setae protruding from raised bumps on the surface, with fold-like microstructures on the plant leaf beetle elytra *Anomala* sp.

The native elytral surface of the plant leaf beetle *Anomala* sp. consists of regularly spaced cracked folds with an elongated orifice of ca 5.50 µm in length (Figs. 2a, b). Setae (length = 20.52±1.55 µm, diameter at centre = 3.30±0.07 µm) are located on tips of sparsely distributed surface bumps or protrusions (density of 43.08 nm^−2^). They are bent downward (ca. 90°) along the protrusion profile (Fig. 2c) with no evidence of nanostructuring. On the protrusion surface some small scale structuring of square/rectangular features (Fig. 2d) indicates the presence of a wax cover.

**Figure 3:**
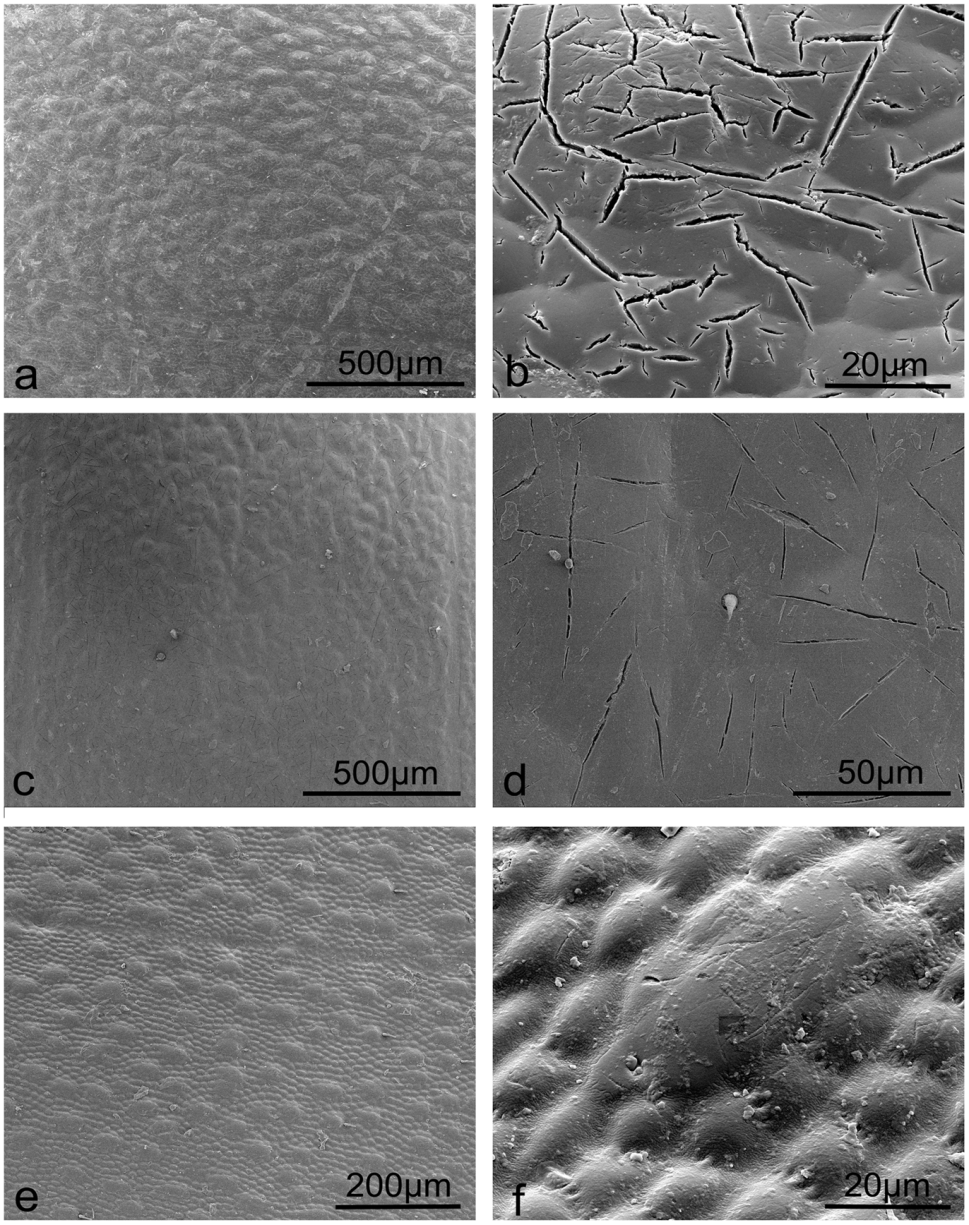
SEM images revealing micro cracks and bumps on the dung beetle elytral surfaces. a, b. *C. molossus*; c, d. *Catharsius* sp.; e, f. *Gymnopleurus* sp.

Among the three dung beetles, *C. molossus* and *Catharsius* sp. possess original elytra with corrugated structures (or bumps) as seen in Figs.3a and c, respectively. The elytral surfaces of both beetles also reveal small cracks (Figs. 3b, d) with a sparse distribution of setae found on sp. 2 (Fig. 3d). The elytral surface of the *Gymnopleurus* sp. has a distribution of larger and smaller elliptical bumps as shown in Fig. 3e and a higher magnification image in Fig. 3f.

**Figure 4:**
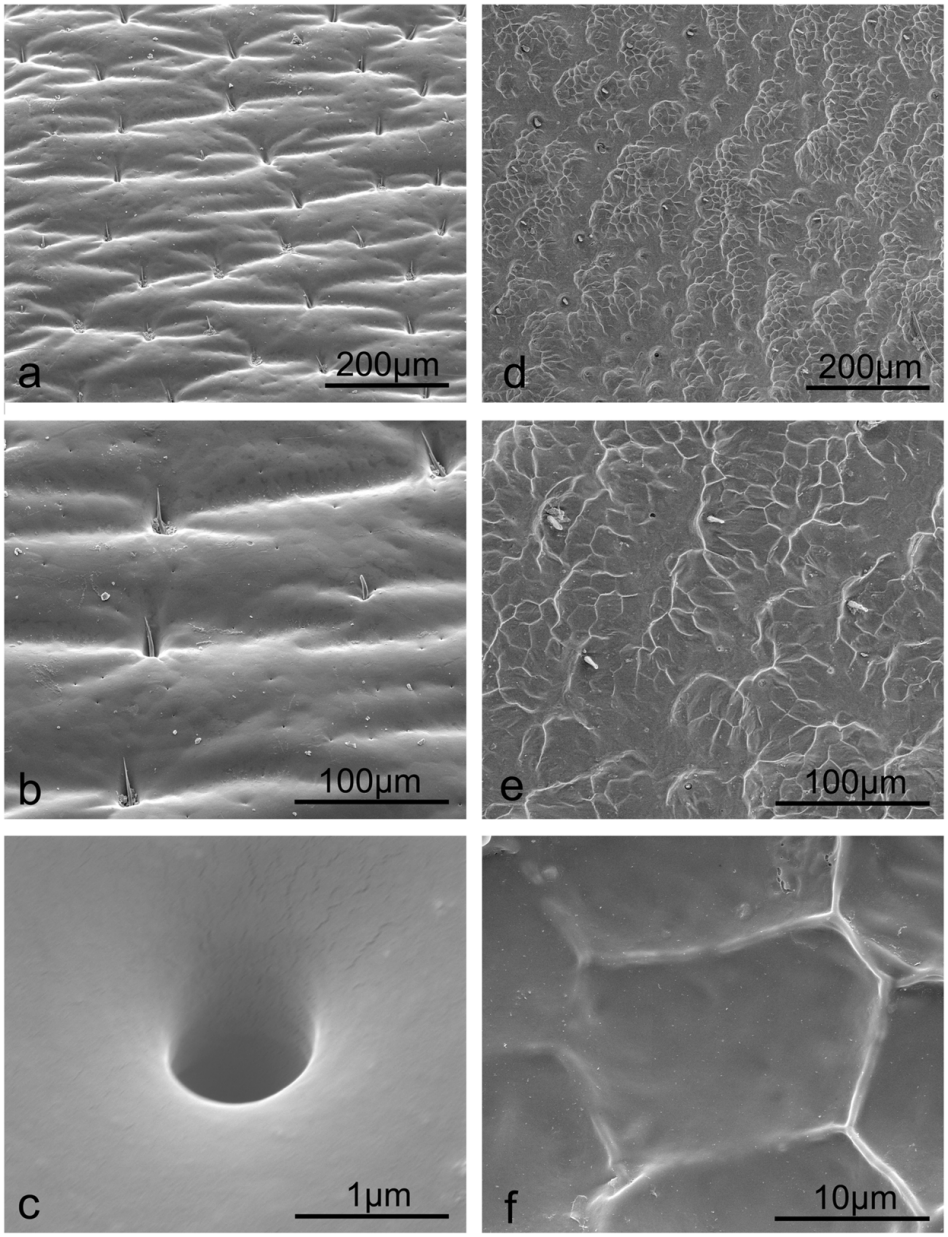
SEM imaging microstructures, setae and secretion pores of the semi-aquatic beetle elytral surfaces. a–c. *S. macrocnemia*; d–f. *A. sinica*.

The native elytra of the two semi-aquatic beetles studied were found to be relatively rough compared to the three aquatic beetles (Table 1). The *S. macrocnemia* beetle elytral surface shows regular corrugations (Fig. 4a), with setae (length = 32.21±1.76 µm and diameter at centre = 2.75±0.16 µm) distributed in the cavities (Fig. 4b) and oriented flat against the elytral surface. The pores (diameter = 0.95 µm) were found to be simple (Fig. 4c). The elytral surface of *A. sinica* exhibits a semi-ordered structuring at low magnification (Fig. 4d) with a sparse setae and pore distribution (Fig. 4e). A polygonal patterning was revealed with an image magnification of two thousand or more (Fig. 4f).

**Figure 5:**
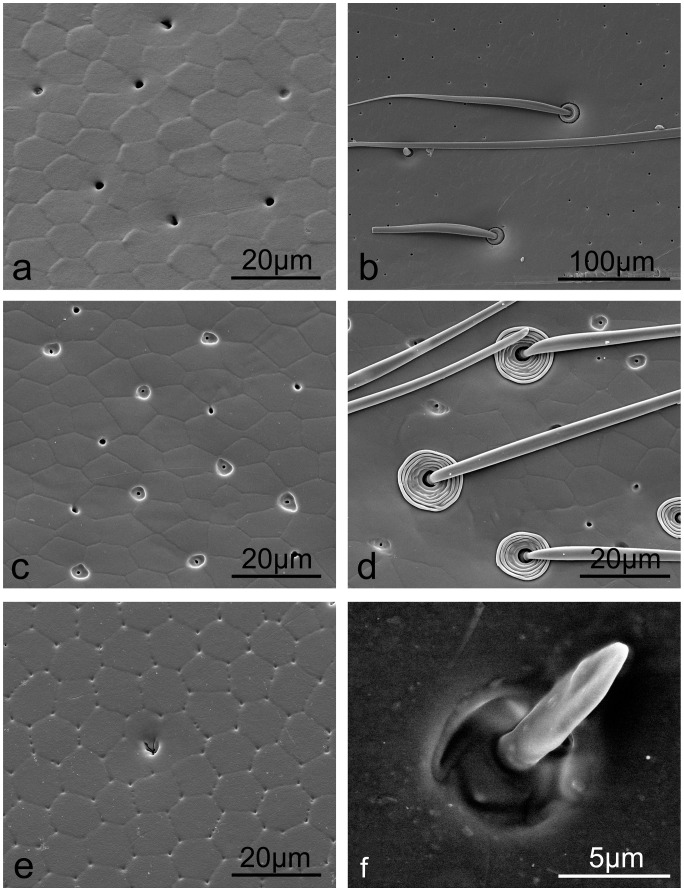
SEM images of aquatic beetle elytra show hexagonally-shaped scale like microstructures, secretion pores and setae of various dimensions. a, b. *H. dauricus*; c, d. *H. grammicus*; e, f. *Hydrochara* sp.

The original elytra of three aquatic beetles revealed quadrangular, pentagonal and hexagonal structuring (*H. dauricus* Fig. 5a, *H. grammicus* Fig. 5c and *Hydrochara* sp. Fig. 5e). The pores on the *H. dauricus* elytra are abundant and simple in structure (diameter = 1.93 µm) (Fig. 5a), whilst on *H. grammicus*, two types of pores coexist; one (diameter = 1.53 µm) structurally simple and similar to that of the *H. dauricus* elytra, and the other (diameter = 0.89 µm) embedded in an expanded and irregular depression (Fig. 5c). The pores found on *Hydrochara* sp. are decorated with a flowering orifice as seen in Fig. 5e. All three species possess variously structured and shaped setae on the elytra. *H. dauricus* and *H. grammicus* reveal thin and long setae (diameter at centre = 9.19±1.41 µm and 2.71±0.36 µm, and length of 533.33±57.74 µm and 127.28±6.82 µm, respectively) with a basal doughnut shaped and concentric circle decorated socket as seen in Figs. 5b and d, respectively. The setae in *Hydrochara* sp. are comparatively short in relation to the other aquatic species (length = 6.43±1.77 µm and diameter at centre = 1.63±0.50 µm) protruding from a relatively simple socket (Fig. 5f).

**Figure 6:**
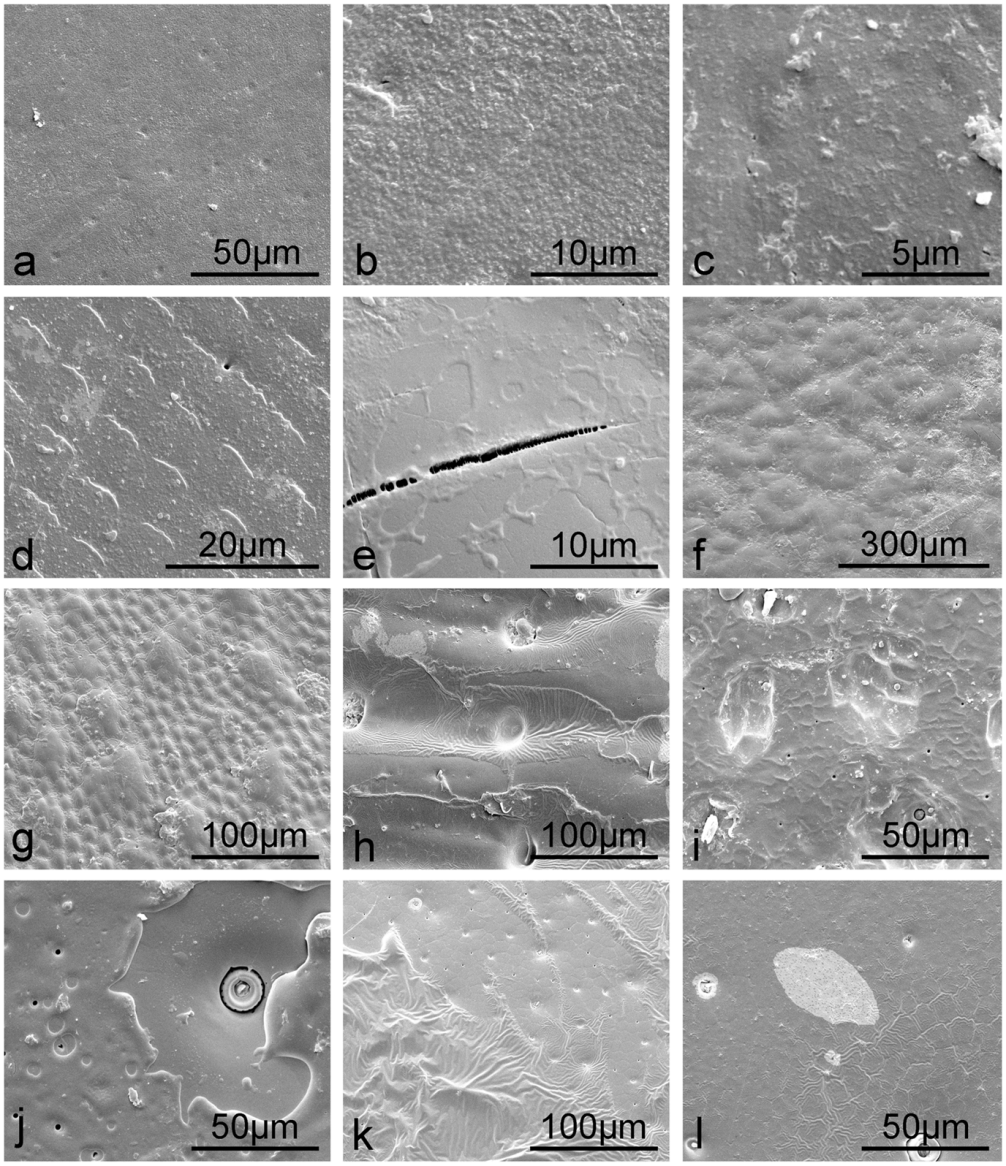
SEM images of elytral surfaces after rinsing with flowing chloroform revealing a layer of wax. a, b. *A. kulzeri*; c. *M. semenowi*; d. *Anomala* sp.; e. *C. molossus*; f. *Catharsius* sp.; g. *Gymnopleurus* sp.; h. *S. macrocnemia*; i. *A. sinica*; j. *H. dauricus*; k. *H. grammicus*; l. *Hydrochara* sp.

After the flowing chloroform treatment, a layer of substance was found on all of the elytral surfaces (Fig. 6). The dissolved substances were evaporated with chloroform on the higher regions (compared to the pits or troughs) of the microstructures (Figs. 6a–i) or still preserved *in situ* on the elytral surfaces (Figs. 6h–l). Accordingly, the surfaces become rougher than that of native elytra (Table 1).

### CA Measurements

Through the examination of water droplets on the native beetle elytra, the static CAs display a range of 47.5° to 109.1° as shown in Fig. S2 and Table 1. The elytra of the desert beetles are hydrophilic with CAs of 47.5° and 78.8° for *A. kulzeri* (Fig. S2a) and *M. semenowi* (Fig. S2d), respectively. The plant leaf beetle, *Anomala* sp., with a CA of 89.9° (Fig. S2g) is in the demarcation point of hydrophilicity and hydrophobicity. The three dung beetles, *C. molossus*, *Catharsius* sp. and *Gymnopleurus* sp., show different wettabilities, ranging from hydrophobic to hydrophilic properties with CAs of 106.9° (Fig. S2i), 93.9° (Fig. S2h) and 71.3° (Fig. S2c), respectively. The two semi-aquatic species of *S. macrocnemia* and *A. sinica* show slightly higher CAs of 107.5° (Fig. S2j) and 109.1° (Fig. S2k), respectively. However, all the three water dwelling beetles, *H. dauricus*, *H. grammicus* and *Hydrochara* sp., exhibit hydrophilic properties with CAs of 66.2° (Fig. S2b), 79.9° (Fig. S2e) and 88.3° (Fig. S2f), respectively.

In contrast, after flowing chloroform treatment, all the CAs on the elytral surfaces increased. The minimal difference of 0.9° between the untreated and treated elytral surface was found on the semi-aquatic beetle *A. sinica*. On the other hand, the maximum difference of 41.1° was found on the surface of the dung beetle *Gymnopleurus* sp. elytra. It was found that almost all the hydrophilic elytra surfaces, with the exception of *A. kulzeri*, become hydrophobic. The CA values of *M. semenowi*, *Anomala* sp., *Gymnopleurus* sp. and the three aquatic beetles all increased to 105.8°, 112.2°, 112.4°, 97.6°, 105.1° and 103.1°, respectively. The hydrophobic elytra of the two dung beetles, *C. molossus* and *Catharsius* sp., and the two semi-aquatic beetles increased to CAs of 114.8°, 105.0°, 122.1° and 110.0°, respectively (Table 1).

### XPS Analysis

**Figure 7:**
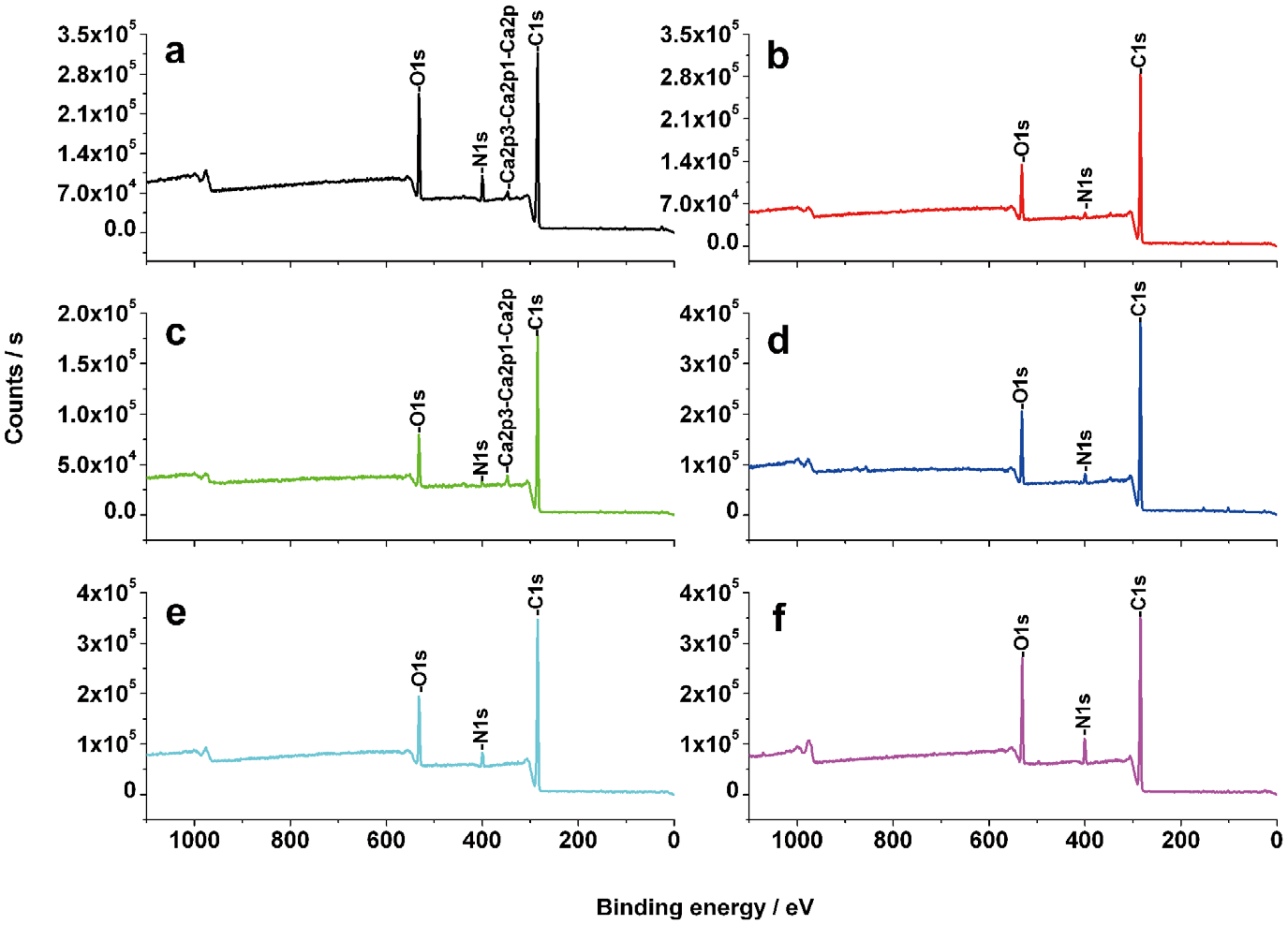
X-ray photoelectron spectroscopy of six native elytral surfaces. a. *A. kulzeri*; b. *Anomala* sp.; c. *C. molossus*; d. *S. macrocnemia*; e. *A. sinica*; f. *H. dauricus*.

Chemical components of six species of beetle elytral surfaces were analyzed by XPS. As shown in Table 2, a total of nine elements, carbon (C), oxygen (O), nitrogen (N), silicon (Si), calcium (Ca), phosphorus (P), sulphur (S), sodium (Na) and aluminium (Al), were identified (Figs. 7, S3). All six beetles were found to contain C, O, N and Si. Examples of Ca and S were present in all samples with the exception of *H. dauricus*. P and S were absent on *C. molossus* elytra and only Na was found on the elytra of *A. sinica* and *H. dauricus*. Traces of Al were found on the elytra of *A. kulzeri* and *S. macrocnemia* (Table 2).

**Table 2:** The chemical contents of six species of beetle elytral surfaces including atom content (a. c.), binding energy (b. e.) and valence state (v. s.) of elements.

Elements	ChemicalContent	Species					
		*A. kulzeri*	*Anomala* sp.	*C. molossus*	*S. macrocnemia*	*A.sinica*	*H.dauricus*
C1s	a. c.	73.09	83.11	84.08	80.31	80.74	80.07
	b. e.	284.8	284.83	284.81	284.83	284.77	284.81
	v. s.	C-C, C-H	C-C, C-H	C-C, C-H	C-C, C-H	C-C, C-H	C-C, C-H
O1s	a. c.	17.09	12	9.87	13.09	12.54	13.24
	b. e.	531.82	532.23	531.74	532	531.94	531.44
	v. s.	O-S	O-S	O-H, O-S	O-S	O-S	O-H, O-S
N1s	a. c.	6.31	2.35	2.04	2.96	4.23	4.88
	b. e.	399.9	400.1	400.28	399.97	399.83	400.9
	v. s.	N-C	N-C	N-C	N-C	N-C	N-C
Si2p	a. c.	1.02	1.2	2.19	1.9	0.88	0.86
	b. e.	102.29	102.14	101.89	102.04	101.91	102.03
	v. s.	Si-O, Si-N	Si-O, Si-N	Si-C	Si-O, Si-N	Si-C	Si-C
Ca2p	a. c.	1.07	0.51	1.81	0.59	0.46	–
	b. e.	347.38	347.19	347.3	347.37	347.1	–
	v. s.	Ca-O	Ca-O	Ca-O	Ca-O	Ca-O	–
P2p	a. c.	0.44	0.65	–	0.5	0.47	0.42
	b. e.	133.55	133.4	–	133.47	133.23	133.36
	v. s.	P-O	P-O	–	P-O	P-O	P-O
S2p	a. c.	0.39	0.18	–	0.35	0.37	–
	b. e.	168.2	168.26	–	167.92	168.22	–
	v. s.	S-C, S-O	S-C, S-O	–	S-C, S-O	S-C, S-O	–
Na1s	a. c.	–	–	–	–	0.31	0.53
	b. e.	–	–	–	–	1071.07	1070.83
	v. s.	–	–	–	–	Na-O	Na-N
Al2p	a. c.	0.59	–	–	0.3	–	–
	b. e.	74.3	–	–	73.74	–	–
	v. s.	Al-O	–	–	Al-O	–	–

**Footnote:** A dash (–) indicates the elements on the elytral surfaces are absent.

Despite the similar peak characteristics of these elements (Fig. S3a), the percentages in atom content (a. c.) were different among individual samples (Table 2). The strongest characteristic photoelectron peak at the binding energies (b. e.) of ca. 285 eV showed the element C to be the main component, the percentage of carbon (C) was highest on the surface of *C. molossus* (84.08) followed by *Anomala* sp. (83.11), *A. sinica* (80.74), *S. macrocnemia* (80.31), *H. dauricus* (80.07), and lowest on *A. kulzeri* (73.09). At the binding energies of ca. 532 eV, 400 eV and 102 eV, the weak peaks denoted the elements O, N and Si, respectively. A small amount of P and S on the wing surfaces were found at about 133 eV and 168 eV, respectively. Furthermore, three metallic elements Ca, Na and Al were also found at ca. 347 eV, 1071 eV and 74 eV. *A. kulzeri* contained the highest content of O and N on the elytral surface (17.09 and 6.31, respectively), whereas the elements Si (2.19) and Ca (1.81) were the most concentrated on the surface of *C. molossus* (Table 2).

### Surface Roughness

**Figure 8:**
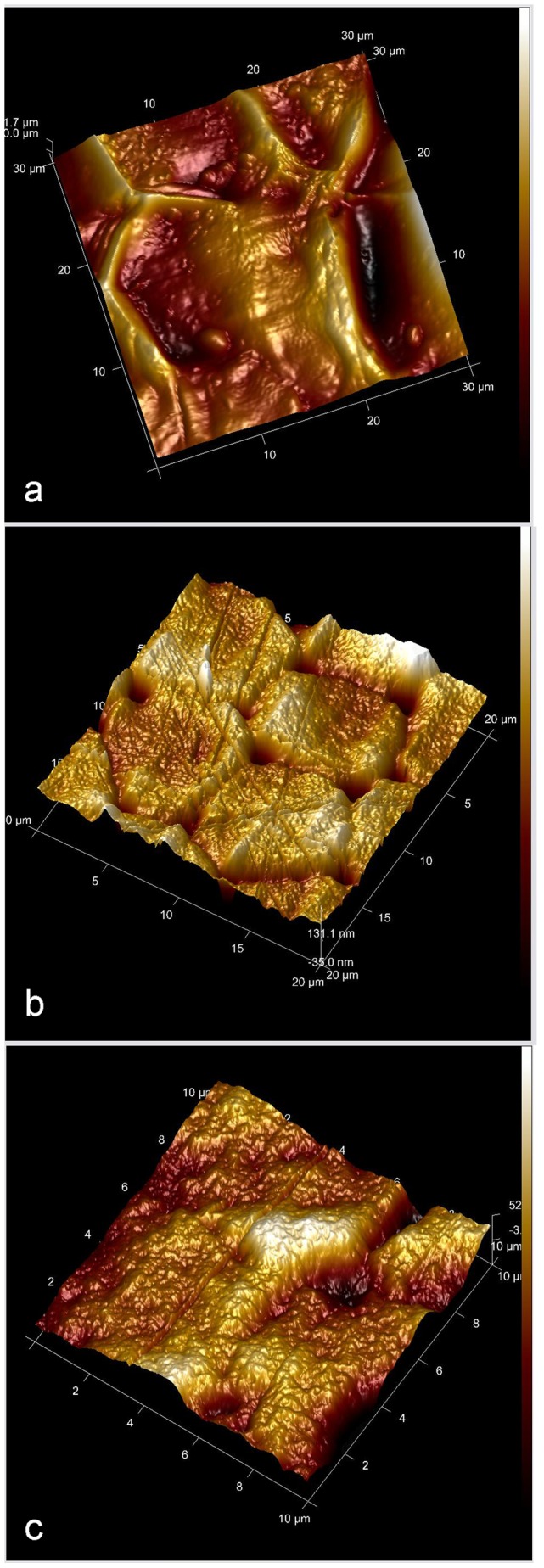
AFM images of scanning range 20 µm^2^ on three native elytral surfaces. a. *A. sinica*, semi-aquatic beetle, the root mean square (RMS) roughness is 148 nm; b. *Hydrochara* sp., aquatic beetle, the RMS is 17.4 nm; c. *H. dauricus*, aquatic beetle, the RMS is 5.94 nm.

The RMS values of the three native elytra of *H. dauricus*, *Hydrochara* sp. and *A. sinica* are 5.94, 17.4 and 148 nm (Fig. 8), respectively. The Ra values of all elytra after choloform treatment range from 64×10^2 ^nm to 154×10^2 ^nm, which are higher than the native elytra of 31×10^2 ^nm on *M. semenowi* to 141×10^2 ^nm on *C. molossus*. The gradual increase in Ra values of the native elytra of *H. dauricus*, *Hydrochara* sp. and *A. sinica* are in accordance with the RMS values obtained using AFM, of 37, 52 and 118×10^2 ^nm, respectively.

## Discussion

The elytra of the 11 species of beetles studied exhibited different wettabilities dependent on the structure, chemistry and environment. Relationships between these factors are analyzed below.

### Relationship between Microstructure and Wettability

The two desert beetles were found to possess hydrophilic properties owing to the seemingly smooth native surfaces. The setae of the two desert species in our study are not dense enough to enhance hydrophobic properties [36]. The nanochannels (Fig. 1e) along the hair shaft of *M. semenowi* setae may be involved in channeling of water. They may also be an evolutionary remnant of superhydrophobicity [20]. Compared to *A. kulzeri*, with a CA of 47.5° (Table 1, Figs. S1a, S2a), the more prominent wax cover (Figs. 6b, c) may play a role in the larger CA of 78.8°of *M. semenowi* (Table 1, Figs. S1b, S2d) [15]. After being rinsed with chloroform, though both surfaces become rougher than native surfaces (Table 1), the majority of the surface material on *A. kulzeri* washed off with flowing chloroform (Figs. 6a, b). The Ra value of 64×10^2 ^nm, however, is not large enough to obtain a change from hydrophilic to hydrophobic, (even though the CA increased by 33.2°). Whilst the wax layer on the *M. semenowi* elytra was preserved (Fig. 6c), the higher Ra (91×10^2 ^nm) enhanced the hydrophobic properties, increasing from 78.8° to 105.8°.

The tapered protrusions on the native elytra of the plant leaf beetle *Anomala* sp. (Fig. 2) may enhance hydrophobicity as it increases the surface roughness, however, the low density of these structures, as with the desert beetle, will be insufficient to resist penetration by larger water droplets (e.g., µL volumes) or smaller droplets resting between structuring. Also, the setae will enable water to slide easily onto the composite surface. The observed elongated cracks are most likely related to the beetles’ coloration [34] rather than contribute to hydrophobicity. All these structures result in the weakly hydrophilic elytra with a CA of 89.9° (Table 1, Figs. S1c, S2g). The wax particles (Fig. 6d) increased the CA to 112.2° (Table 1) on the elytral surface after chloroform treatment owing to the Ra increasing from 65×10^2 ^nm to162×10^2 ^nm.

The wetting properties of the three dung beetles’ native elytra, *C. molossus*, *Catharsius* sp. and *Gymnopleurus* sp., differs with the first two being hydrophobic (106.9° and 93.9°, Table 1, Figs. S1d, S2i and S1e, S2h, respectively), while *Gymnopleurus* sp. presented a hydrophilic elytra with a CA of 71.3° (Table 1, Figs. S1f, S2c). The greatest Ra value (141×10^2 ^nm) resulting from a higher density of micro-cracks (Fig. 3b) and which may reduce the water contact area, may also contribute to a greater CA (106.9°) and an increase in hydrophobic properties on the elytra of *C. molossus*. Despite a higher Ra value (104×10^2 ^nm) on the *Gymnopleurus* sp. elytra, the biggest contact area of elliptical bumps with water leads to the lowest CA of 71.3°. One could expect on the *Catharsius* sp. elytra, with a smaller Ra value (47×10^2 ^nm), that the chemistry should be partly responsible for the hydrophobicity (with a CA of 93.9°). After the chloroform treatment however, the increased values of CAs on the elytral surfaces of *C. molossus* and *Catharsius* sp. (differences of 7.9° and 11.1°, respectively) were much smaller indicating that the surface cracks mainly determine the hydrophobicity. The wax particles tended to accumulate around the rims of the larger bumps of *Gymnopleurus* sp. (see Fig. 6g) increasing the surface roughness (132×10^2 ^nm) and thus increasing the CA from 71.3° to 112.4°.

The two semi-aquatic beetles studied achieve hydrophobic properties by means of rough structures on their native elytra. *S. macrocnemia* presents a wavy surface structure with setae distributed between ca. 50 to 250 µm apart (Figs. S1g, 4a). *A. sinica* on the other hand, presents a semi-ordered surface structure on their elytra. Both insects present different surface structuring which enhances the roughness of the elytra and thus results in higher CAs (107.5° and 109.1°) and hydrophobic properties [37]. The Ra value of *S. macrocnemia* is lower (74×10^2 ^nm) when compared to *A. sinica* (118×10^2 ^nm). The CA value however is nearly equal to the latter indicating that in enhancing hydrophobicity, chemistry also plays an important role. Chloroform treatment enhanced both the surface hydrophobicity (CA values increased by 14.6° on *S. macrocnemia* elytra and only 0.9° on the surface of *A. sinica*) and the RA values (increase of 61×10^2^ nm on *S. macrocnemia* elytra and 23×10^2^ nm on *A. sinica*) of both the semi-aquatic species. As with *C. molossus* and *Catharsius* sp. dung beatles, the rough surface microstructures are the main reason of improving CAs.

The native elytra of three aquatic beetles (*H. dauricus*, *H. grammicus* and *Hydrochara* sp.) show comparatively smoother surfaces (Ra values of 37, 49 and 52×10^2 ^nm, respectively) in comparison with the semi-aquatic insects. Their CAs are also lower, all <90° (66.2°, 79.9° and 88.3° as shown in Table 1 and Figs. S2b, e and f, respectively). Their seemingly smoother surfaces can be attributed to their confines of living in an aquatic environment. As Fig. 8 shows, the roughness values of *H. dauricus* and *Hydrochara* sp. are only 5.94 (Fig. 8c) and 17.4 nm (Fig. 8b), respectively, compared to the higher roughness of *A. sinica*, 148 nm (Fig. 8a). Additional contributions can be attributed (in part) to secretions through the pore channels [38]. From Fig. S4, it can be seen that the formation of menisci at the interface between elytra of *H. dauricus* and water during sliding contact resulted from wetting, which increased adhesion and friction [39]. The water droplet adheres to the elytral surface of *H. dauricus* and remains pinned even when the plate is titled to 90°. This indicates that the mechanism of water interacting with the elytra is not as a result of elytral surface roughness alone. A possible explanation is related to hydrokinetics. After immerging in water, a layer of flowing water film is formed on the elytra surface and is in a state of dynamic balance between wing surface and the fluid. Further studies are required to fully interpret the mechanism for this water pinning of the aquatic beetle elytra. After chloroform treatment, all aquatic beetle elytra changed from hydrophilic (CAs = 66.2°, 79.9° and 88.3°) into hydrophobic (CAs = 97.6°, 105.1° and 103.1°) and the elytra Ra values increased (104, 79 and 89×10^2 ^nm).

### Relationship between Chemistry and Wettability

The cuticle of most insects is covered by lipoids consisting chiefly of hydrocarbons and esters, which are solid waxes forming a layer approximately 0.25 µm thick on the epicuticle [38]. The water-proofing abilities of the cuticle depends upon the physical properties (including chain length, unsaturation and methyl-branching), which depend in turn upon their chemical composition [40]. According to the binding energy of examined elements (Table 2), the valence states (v. s.) can be determined using the Handbook of X-ray Photoelectron Spectroscopy [41] and the NIST X-ray Photoelectron Spectroscopy Database [42]. Carbon (C) should originate from protein, wax or phenolic compounds, oxygen (O) from hydroxyl groups and the oxidation of sulphur, and sulphur (S) from amino acids. Phosphorus (P) should originate from phospholipids, though they are rarely found on the surface of arthropods, and their presence may result from contamination from internal membranes [43]. However, the origins of the silicon (Si), calcium (Ca), sodium (Na) and aluminium (Al) were unclear from the experimental data. It is likely that the silicon (Si) and calcium (Ca) participated in the formation of the surface crystal structure. Sodium (Na) and aluminium (Al) on the other hand, may possibly mainly function as a role of regulating the acid and alkali balance.

The secondary structure of protein side chains can be blocked, so we focused only on the surface functional groups. The long-chain hydrocarbons, typically ranging in length from 21 to >40 carbons and often containing one or more double bonds or methyl branches, are the predominant constituents. Oxygenated lipids such as wax esters and ketones also occur [43]. However, it’s well known that typically both microstructure and chemistry jointly determine the wettability of solids. The presence of a wax cover alone (Figs. S5a, b) cannot determine hydrophobic properties, but rather combined with the microstructures and secretions (Fig. S5c). As shown in Fig.6, after treatment with flowing chloroform, the wax was found on all of the elytral surfaces. Their specific components however should be different among them due to their different solubility and final state. On the desert beetles the wax almost completely dissolved after chloroform treatment (Figs. 6a, b), while on the aquatic beetles almost all of the wax was preserved *in situ* (Figs. 6j–l). This is confirmed by X-ray spectra (shown in Fig. S3).

### Relationship between Aging and Wettability

Aging (referring to the time after emergence) mainly affects the hydrophobic properties of insect cuticle through changing of the microstructures and chemistry of samples, which are the main effecting factors on the wettability of solid surfaces.

On the well-developed samples, the surface microstructures of elytra may not change significantly over time. The structural characteristics such as dimensional properties, furrow strip and concavo-convex [33], polygonal pattern and parallel ridges [16] of the dry samples of *C. molossus* are very similar to the fresh samples of *Copris ochus* [32]. However immediate emergent samples, such as *A. sinica* (teneral individuals), appeared yellowish-brown (aged specimens are black in color) [44]. When the sample is fresh, the elytral surfaces should display punctate striae compared to the depressed structuring in this study (Figs. 4d–f). So the aging has a significant influence on the microstructure of newly emerged species. This is mainly due to the surface of freshly emerged samples not being completely tanned, thus the surface cannot function as a shield to prevent water evaporating, the CAs on the elytral surfaces before and after chloroform treatment were found to be very similar, 109.1° and 110.0°, respectively.

During the progress of natural desiccating of insect wings, dehydration is not expected to change the surface chemistry (energy levels are too low, and enzyme activity is not present) but the co-operative interactions between the proteins will be enhanced (as the change of beta structures). As well the chemical composition of wing tissue cannot be synthesized and added in a steady stream of delivery. This is consistent with XPS data which examines only the outermost cuticle of dried samples.

As for the effect of aging on CA, the newly moulted cuticle is completely hydrophobic. In the first two hours it shows hydrophilic properties, but after four hours becomes persistently hydrophobic again [38]. In this study, a similar result is obtained, where the CA of the same elytra is consistent with the passage of time. CAs changed less than 11° from the initial measurements (see Table S1). So the aging of elytra appears to be less effective on microstructure, chemistry and wettability than their mobile secretions and the corresponding original activity.

### Relationship of Wettability with Other Functions

The elytra of beetles fulfill numerous other functions than just those addressed in the previous section. For example, hairs are often sensors and not only structures influencing wetting properties. Holes and/or cracks may be related to the tensile strength of cellular solid materials [45].

The function of forewings of Coleopteran is of great ecological significance. The forewings of the desert beetles are dorsally held together (note the black line indicating the joint of two elytra highlighted by the red arrow in Fig. 1d) and extend to the ventral side as a shell (Figs. S6a, b) in order to support them whilst crawling in the desert freely. The lateral sides of the thorax, elytra and sternum with round protrusions (marked with red arrows in Figs. S6b, c) may aid in limiting the opportunities of contact with sand. This may also allow reduced contact area and time with potentially extremely hot sand particles which may present a direct threat to their lives [46]. The wings possess hydrophilic properties and may function as a water catchment device to survive in the hot and dry climate [47].

The complex microstructure of the plant beetle *Anomala* sp. contributed to the formation of the green surface coloration as confirmed via reflectance spectra conducted with a fiber-optic spectrometer (UV-VIS-NIR Lightsource DH-2000). As shown in Fig. S7, the position of the reflection peak moved with the change of incident angles. The reflection peak appears at a wavelength of ca. 560 nm when the incident angle is 0°, while at an incident angle of 45° the reflection peak presents at a wavelength of ca. 549 nm. The hydrophilic surface of the elytra enhances the interaction with light [27] allowing it to maintain its green coloration and thus camouflage (Fig. S1c).

Diminishing hydrophobic properties found among the three dung beetles studied resulted in the adhesive and/or frictional forces increasing. The structures found on *Gymnopleurus* sp. consist of large and small protrusions which increase the contact area with water and increase the adhesive force. The *C. molossus* and *Catharsius* sp. beetles present corrugated structures and tiny cracks which may play a role in alteration (lowering) of adhesive forces. In addition, their prothorax surfaces are all rough with rounded or polygonal protrusions (Fig. S8). All of these non-smooth surfaces reduce the contact areas of elytra with their habitat, and thus minimize the friction between the surfaces [32], [48]. The species of genus *Catharsius* are tunnellers, mostly living in grasslands and pastures, occasionally in forests, where they eat large mammal dung and use it to make pedotrophic nests in which their offspring develops. Thus a major function of their elytra is drag- reducing, the structures reduce the opportunity of contacting with moist dung. In the case of the roller *Gymnopleurus* sp., their elytra have a relatively smaller chance of contact with moist dung, so there is no need to evolve additional hydrophobicity.

In contrast to the other beetles, semi-aquatic and aquatic beetles are special groups living in waters during different stages of their life history. Their body sections, structuring and chemistry which make contact with waters should be hydrophobic or hydrophilic in nature depending on the level of immersion in the liquid. Semi-aquatic beetles will present more hydrophobic surfaces (for example *S. macrocnemia* with a CA of 107.5^o^ and *A. sinica* with a CA of 109.1^o^) (Table 1). Fully submerged aquatic beetles should possess weak hydrophobic or hydrophilic chemistry such as *Dytiscus marginalis* (with a CA of 90°) [15]. The elytra of the aquatic species *Hydrobius* sp., has a CA of 87°. Similarly, the diving beetles *Agabus bipustulatus* and *Hydroporus palustris* show the same characteristics of hydrophilicity [16] as *H. dauricus*, *H. grammicus* and *Hydrochara* sp. with CAs of 66.2°, 79.9° and 88.3°, respectively (Table 1).

### Conclusions

From the observation of microstructure, chemistry and wettabilities, we have demonstrated that the same groups of beetle elytra exhibit some consistency in their surface properties in order to exist in their selective environments. All the elytra exhibit compound microstructures. Apart from the chemical nature of the cuticle, irregularities and the presence or absence of tiny cracks, setae (or hairs), pores and protrusions were important in determining the wettability of the surfaces. Generally, the rough elytral surfaces typically demonstrated higher hydrophobicity.

Compared to other beetles, the aquatic beetles have relatively smooth elytral surfaces. These hydrophilic structures provide the beetles with freedom of mobility within the water body. While the roughness of the elytra may reduce contact with water (i.e. water droplets and bulk water bodies), it may also reduce the contact area with solid bodies which the insect may come into contact with (e.g., foliage, sand particles). Reduced contact area will reduce adhesive as well as frictional forces between the contacting surfaces. Understanding the structure-function relationships of the elytra in the context of its physical and biological constraints, may provide optimized parameters for biomimetic materials from specific habitats/environments.

## Supporting Information

Figure S1 Top view photographs of the eleven species of adult beetles studied. a. *Anatolica kulzeri*; b. *Mantichorula semenowi*; c. *Anomala* sp.; d. *Catharsius molossus*; e. *Catharsius* sp.; f. *Gymnopleurus* sp.; g. *Sominella macrocnemia*; h. *Amphizoa sinica*; i. *Hydrophilus dauricus*; j. *Hydaticus grammicus*; k. *Hydrochara* sp. a, b: desert beetles; c: plant beetle; d-f: dung beetles; g, h: semi-aquatic beetles; i-k: aquatic beetles.
(TIF)

Figure S2 Optical images of water droplets on the eleven beetles’ native elytral surfaces. a. *A. kulzeri*, contact angle (CA) = 47.5°; b. *H. dauricus*, CA = 66.2°; c. *Gymnopleurus* sp., CA = 71.3°; d. *M. semenowi*, CA = 78.8°; e. *H. grammicus*, CA = 79.9°; f. *Hydrochara* sp., CA = 88.3°; g. *Anomala* sp., CA = 89.9°; h. *Catharsius* sp., CA = 93.9°; i. *C. molossus*, CA = 106.9°; j. *S. macrocnemia*, CA = 107.5°; k. *A. sinica*, CA = 109.1°.
(TIF)

Figure S3 X-ray photoelectron spectroscopy of six elytral surfaces. a. Full spectra; b. Element C; c. Element O; d. Element N; e. Element Si; f. Element P; g. Element S; h. Element Ca; i. Element Na; j. Element Al.
(TIF)

Figure S4 The adhesion of water droplet on the elytra of *H. dauricus*. a–c. The plate is titled 30°, 60° and 90°, respectively.
(TIF)

Figure S5 SEM images of the beetle elytral surfaces to show the wax cover (a. *Catharsius molossus*; b. *Gymnopleurus* sp.) and the secrete pore (c. *Hydaticus grammicus*).
(TIF)

Figure S6 The lateral and ventral view of desert beetles. a. *A. kulzeri*; b, c. *M. semenowi*. The red arrows show round protrusions of the lateral sides of thorax, elytra and sternum.
(TIF)

Figure S7 The reflectance spectra of elytral surface of the plant leaf beetle *Anomala* sp.
(TIF)

Figure S8 SEM images of three dung beetle prothorax show the rounded or polygonal protrusions. a. *C. molossus*; b. *Catharsius* sp.; c. *Gymnopleurus* sp.
(TIF)

Table S1 Comparable list of contact angles (CAs) measured in different time on the elytral surfaces of four species of beetles inhabiting various environments.
(DOC)
