# Supplementary figures and images for: Compound Microstructures and Wax Layer of Beetle Elytral Surfaces and Their Influence on Wetting Properties
Source: PLoS One. 2012 Oct 4;7(10):e46710. doi: 10.1371/journal.pone.0046710 (PMC3464267; doi:10.1371/journal.pone.0046710)

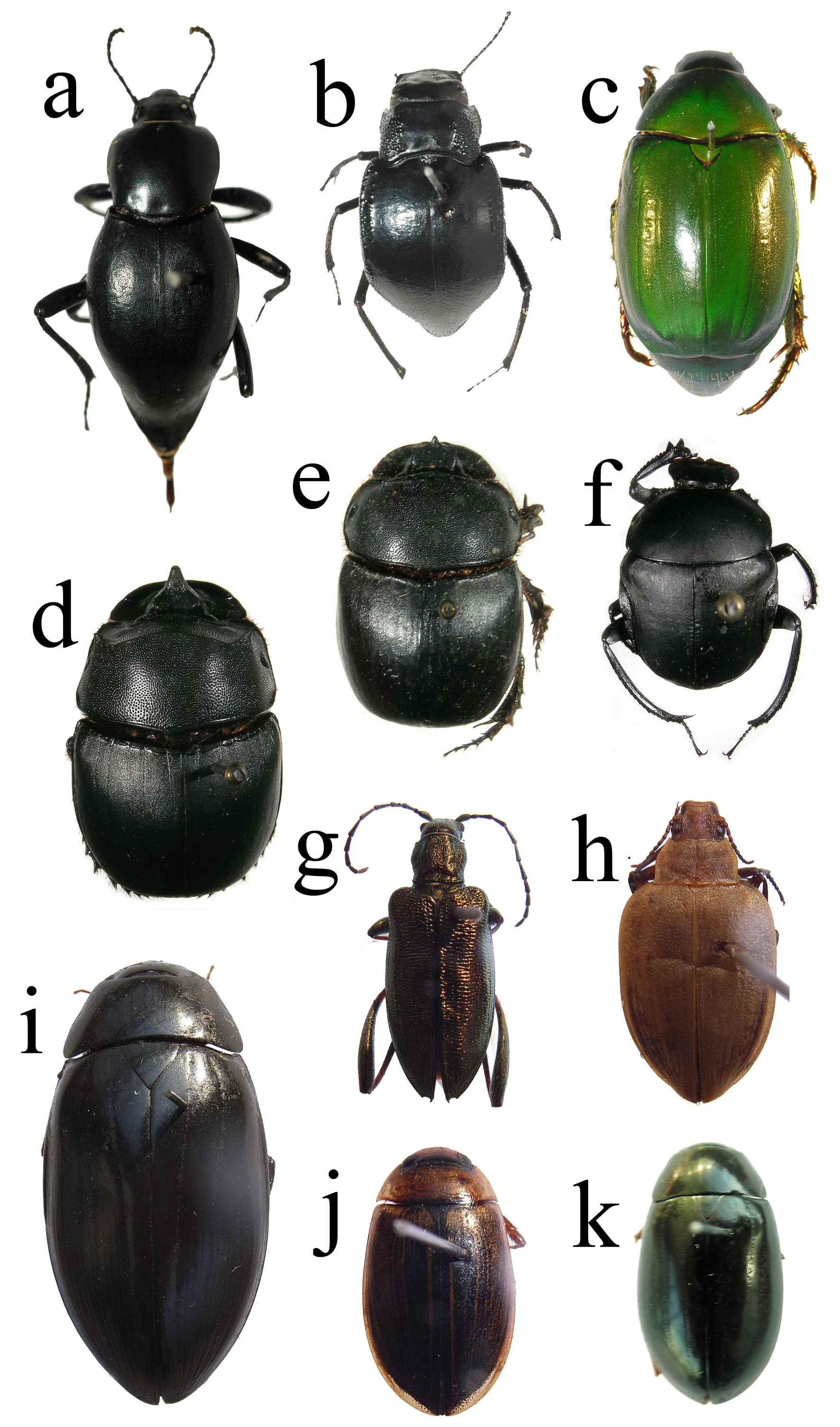

Supplement: Figure S1 — Top view photographs of the eleven species of adult beetles studied. a. Anatolica kulzeri; b. Mantichorula semenowi; c. Anomala sp.; d. Catharsius molossus; e. Catharsius sp.; f. Gymnopleurus sp.; g. Sominella macrocnemia; h. Amphizoa sinica; i. Hydrophilus dauricus; j. Hydaticus grammicus; k. Hydrochara sp. a, b: desert beetles; c: plant beetle; d-f: dung beetles; g, h: semi-aquatic beetles; i-k: aquatic beetles. (TIF) [file pone.0046710.s001.tif]

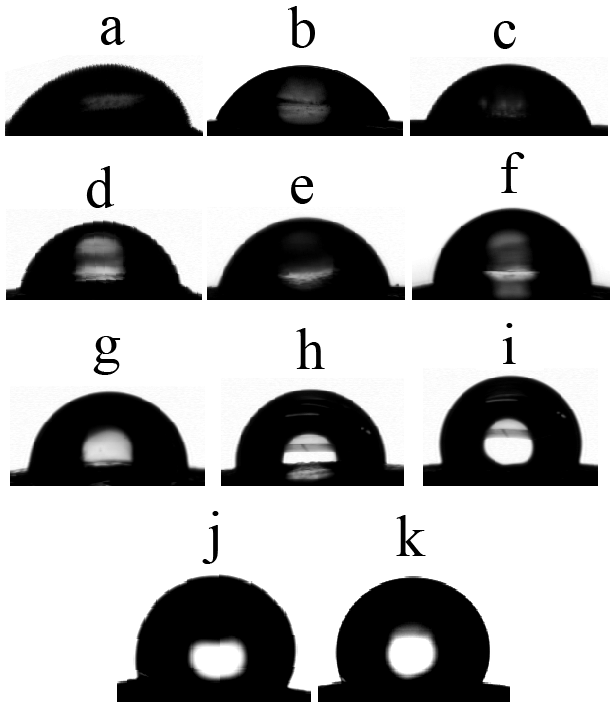

Supplement: Figure S2 — Optical images of water droplets on the eleven beetles’ native elytral surfaces. a. A. kulzeri, contact angle (CA) = 47.5°; b. H. dauricus, CA = 66.2°; c. Gymnopleurus sp., CA = 71.3°; d. M. semenowi, CA = 78.8°; e. H. grammicus, CA = 79.9°; f. Hydrochara sp., CA = 88.3°; g. Anomala sp., CA = 89.9°; h. Catharsius sp., CA = 93.9°; i. C. molossus, CA = 106.9°; j. S. macrocnemia, CA = 107.5°; k. A. sinica, CA = 109.1°. (TIF) [file pone.0046710.s002.tif]

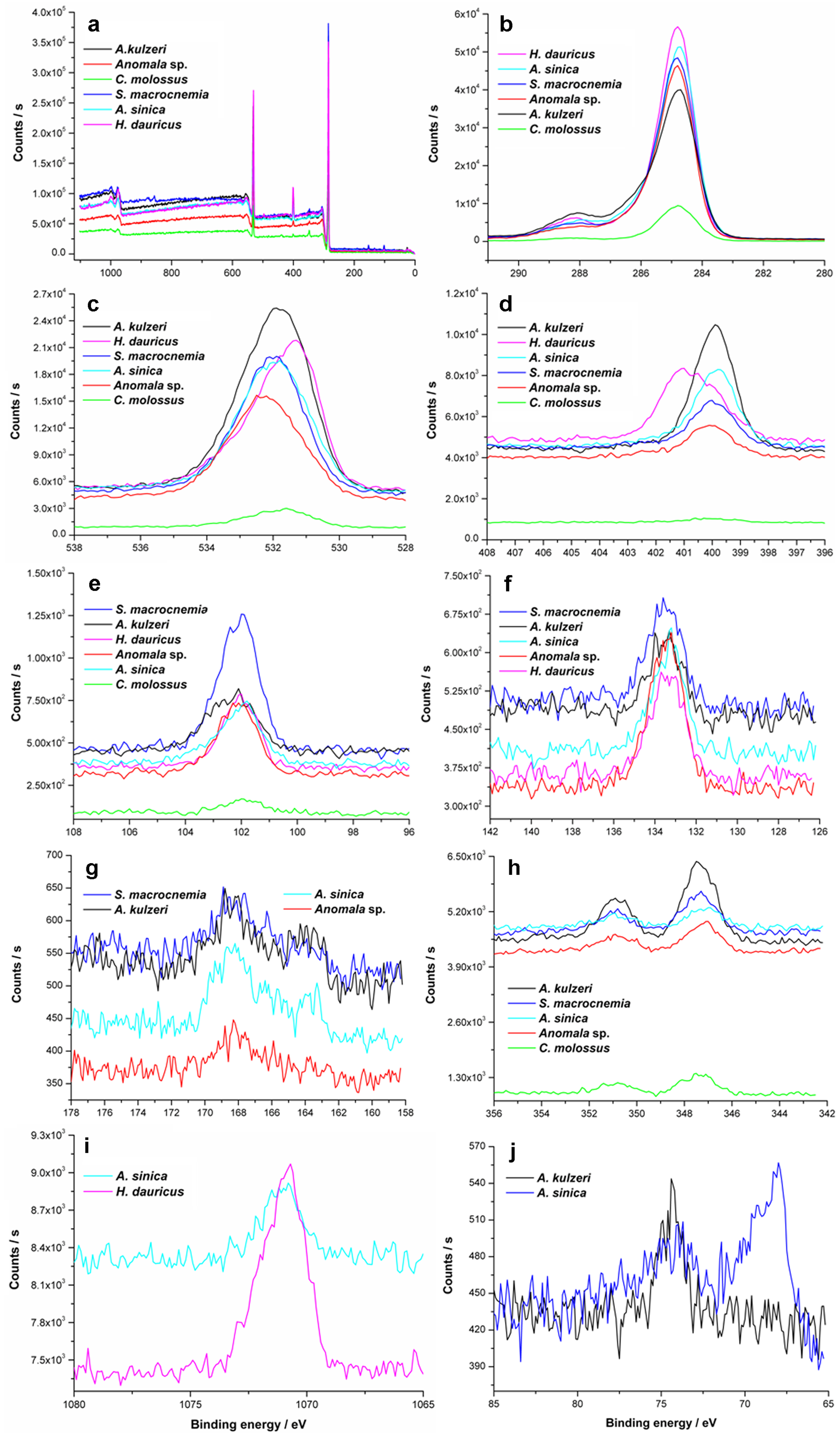

Supplement: Figure S3 — X-ray photoelectron spectroscopy of six elytral surfaces. a. Full spectra; b. Element C; c. Element O; d. Element N; e. Element Si; f. Element P; g. Element S; h. Element Ca; i. Element Na; j. Element Al. (TIF) [file pone.0046710.s003.tif]

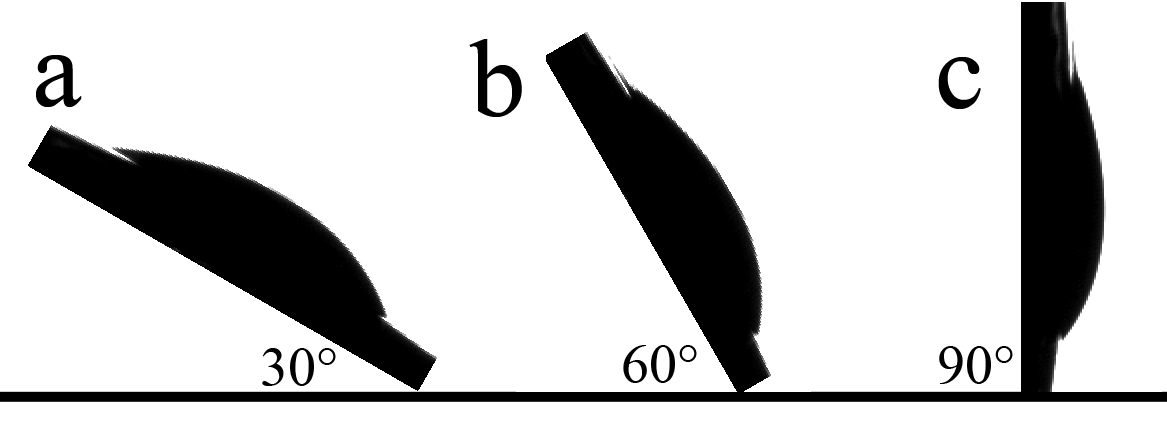

Supplement: Figure S4 — The adhesion of water droplet on the elytra of H. dauricus. a–c. The plate is titled 30°, 60° and 90°, respectively. (TIF) [file pone.0046710.s004.tif]

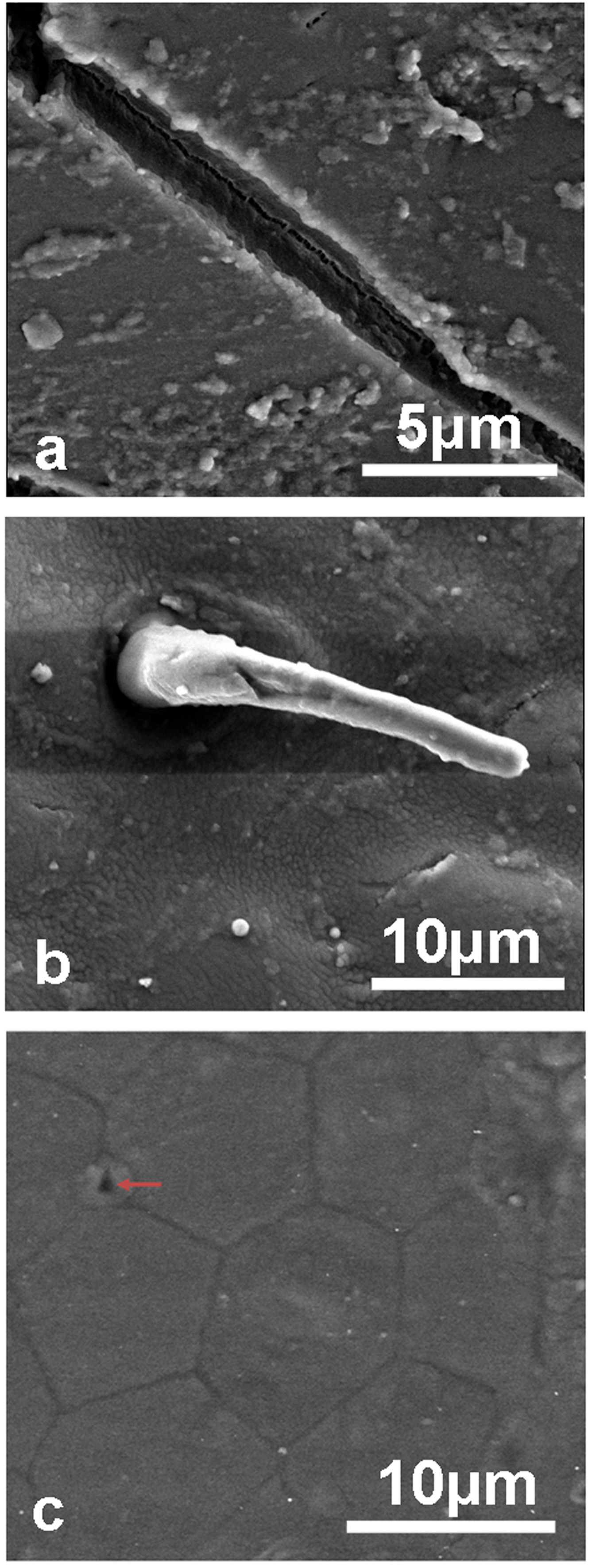

Supplement: Figure S5 — SEM images of the beetle elytral surfaces to show the wax cover (a. Catharsius molossus; b. Gymnopleurus sp.) and the secrete pore (c. Hydaticus grammicus). (TIF) [file pone.0046710.s005.tif]

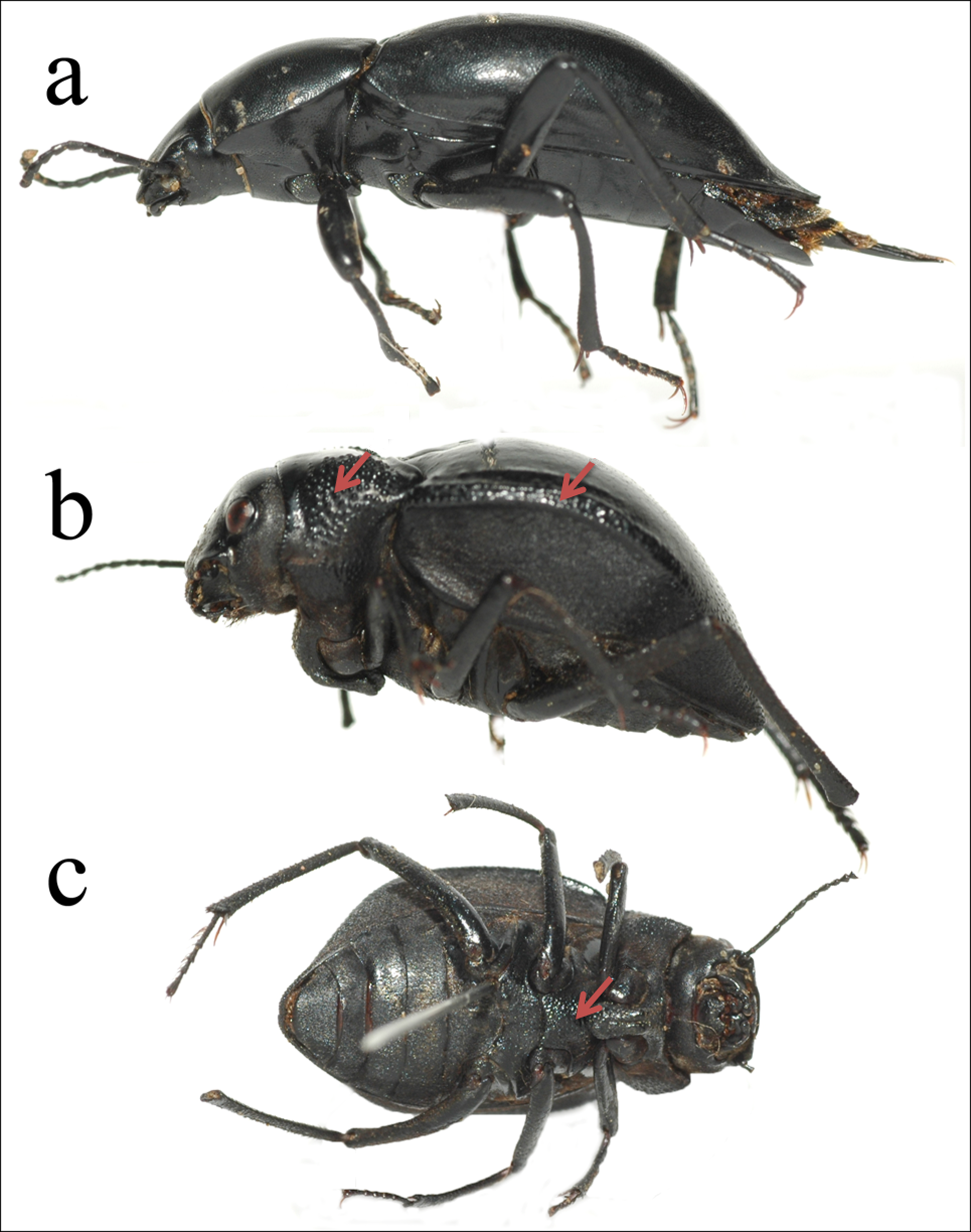

Supplement: Figure S6 — The lateral and ventral view of desert beetles. a. A. kulzeri; b, c. M. semenowi. The red arrows show round protrusions of the lateral sides of thorax, elytra and sternum. (TIF) [file pone.0046710.s006.tif]

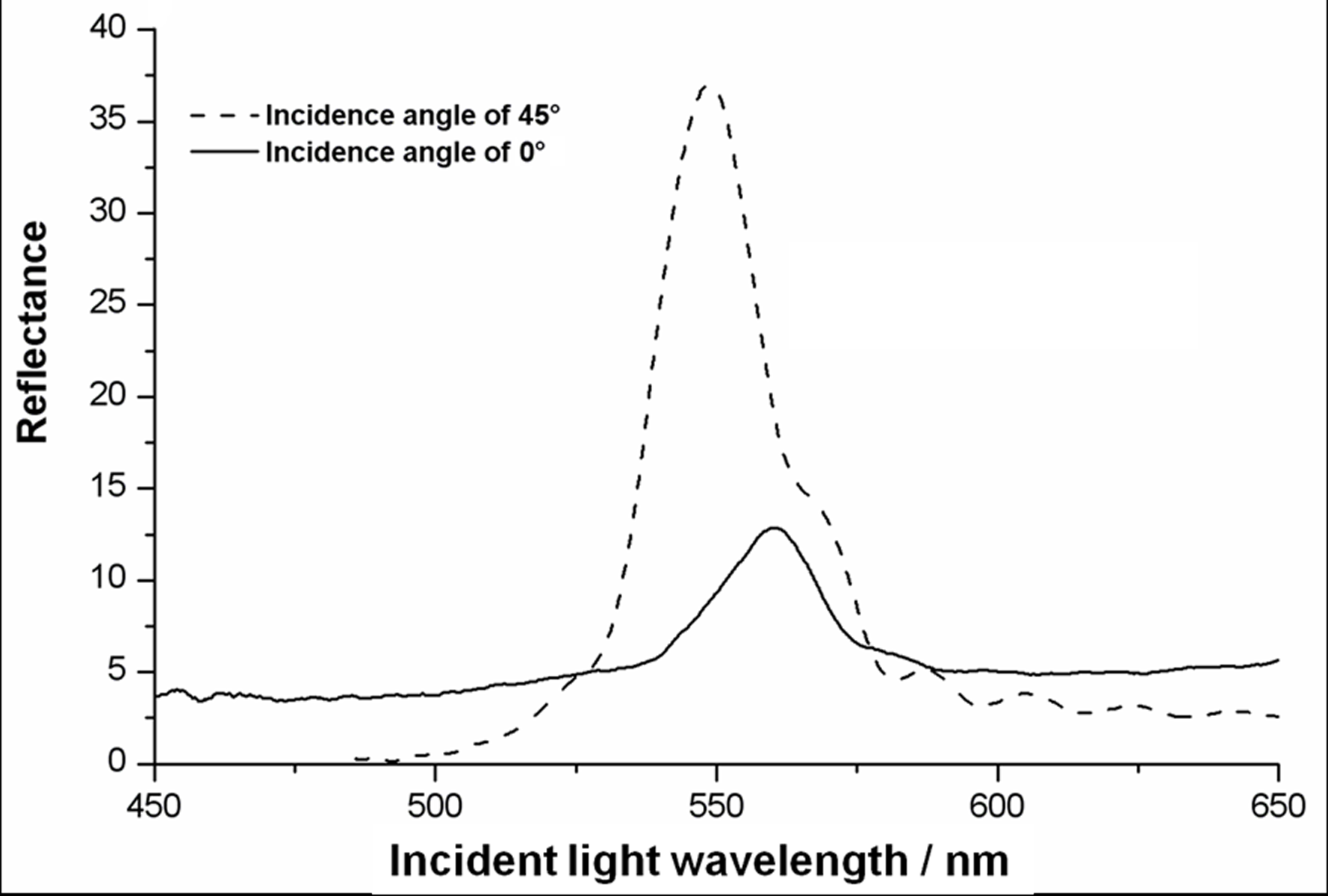

Supplement: Figure S7 — The reflectance spectra of elytral surface of the plant leaf beetle Anomala sp. (TIF) [file pone.0046710.s007.tif]

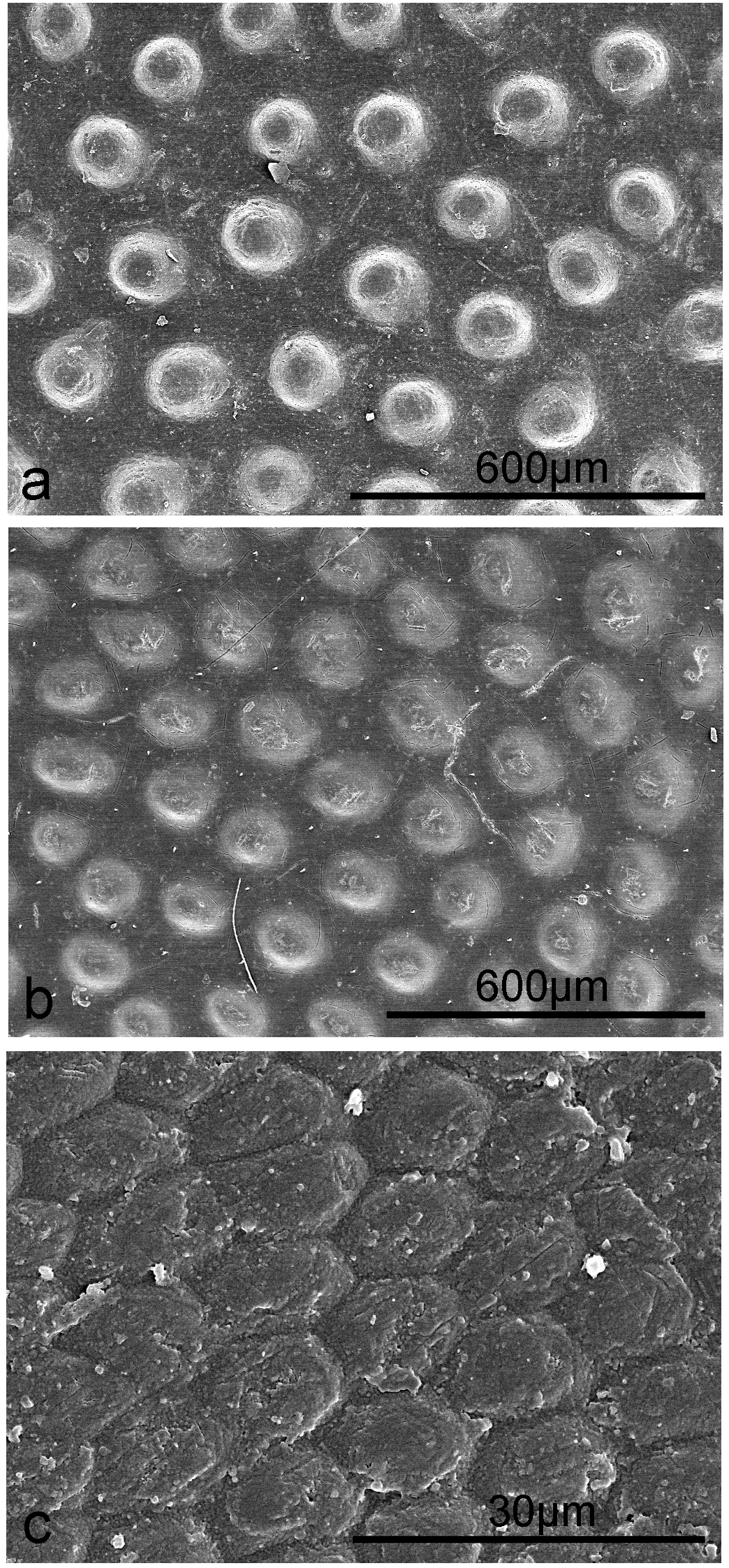

Supplement: Figure S8 — SEM images of three dung beetle prothorax show the rounded or polygonal protrusions. a. C. molossus; b. Catharsius sp.; c. Gymnopleurus sp. (TIF) [file pone.0046710.s008.tif]
