# Supplementary material for: Compound Microstructures and Wax Layer of Beetle Elytral Surfaces and Their Influence on Wetting Properties
Source: PLoS One. 2012 Oct 4;7(10):e46710. doi: 10.1371/journal.pone.0046710 (PMC3464267; doi:10.1371/journal.pone.0046710)
Supplement: Table S1 — Comparable list of contact angles (CAs) measured in different time on the elytral surfaces of four species of beetles inhabiting various environments. (DOC) [file pone.0046710.s009.doc]

**Table S1.** Comparable list of contact angles (CAs) measured in different time on the elytral surfaces of four species of beetles inhabiting various environments.

| **Lables in Fig. S1** | **Species** | **CAs measured in different year (º)** | | **Habitat** |
| --- | --- | --- | --- | --- |
|  |  | **2007** | **2010** |  |
| d | *Catharsius molossus* | 106.9 | 101.8 | dung |
| f | *Gymnopleurus* sp. | 71.3 | 66.8 | dung |
| g | *Sominella macrocnemia* | 107.5 | 114.0 | semi-aquatic |
| k | *Hydrochara* sp. | 88.3 | 99.3 | aquatic |
